# Unique dynamic mode between Artepillin C and human serum albumin implies the characteristics of Brazilian green propolis representative bioactive component

**DOI:** 10.1038/s41598-020-74197-4

**Published:** 2020-10-14

**Authors:** Fan Wu, Xin-Mi Song, Yi-Lei Qiu, Huo-Qing Zheng, Fu-Liang Hu, Hong-Liang Li

**Affiliations:** 1 Zhejiang Provincial Key Laboratory of Biometrology and Inspection & Quarantine, College of Life Sciences, China Jiliang University, Hangzhou, 310018 China; 2 College of Animal Sciences, Zhejiang University, Hangzhou, 310058 China

**Keywords:** Entomology, Mechanism of action

## Abstract

As a representative bioactive component in Brazil green propolis, Artepillin C (ArtC; 3, 5-diprenyl-4-hydroxycinnamic acid) has been reported a wide variety of physiological activities including anti-tumor, anti-inflammatory, and antimicrobial activity etc. However, it seems incompatible that ArtC in vivo was characterized as low absorption efficiency and low bioavailability. In order to obtain the elucidation, we further investigated the physicochemical basis of ArtC interacting with human serum albumin (HSA) in vitro. We found a unique dynamic mode interaction between ArtC and HSA, which is completely different from other reported propolis bioactive components. Thermodynamic analysis showed that hydrophobic interactions and electrostatic forces are the main driving force. The competitive assay indicates that the binding site of ArtC with HSA is close to the Sudlow’s site I. The findings of this study reveal the unique physicochemical transport mechanism of ArtC in the human body, which helps to further understand the uniqueness of the representative functional components of Brazilian green propolis in the human body.

## Introduction

Propolis is a resinous-like material that honey bees collect from various plants exudates and mix them with beeswax to form a sealing material of a certain consistency^1^. As one of the world-famous propolis type, Brazilian green propolis displays as green since it especially comes from *Baccharis dracunculifolia*, a plant resource widely distributed in South America^2–4^. Brazilian green propolis contains a variety of chemical components and is reported to have multiple biomedical activities, such as antibacterial activities, antioxidant, antiulcer, anti-inflammatory, antigenotoxicity and antimutagenicity^5–9^ etc.

**Figure 1:**
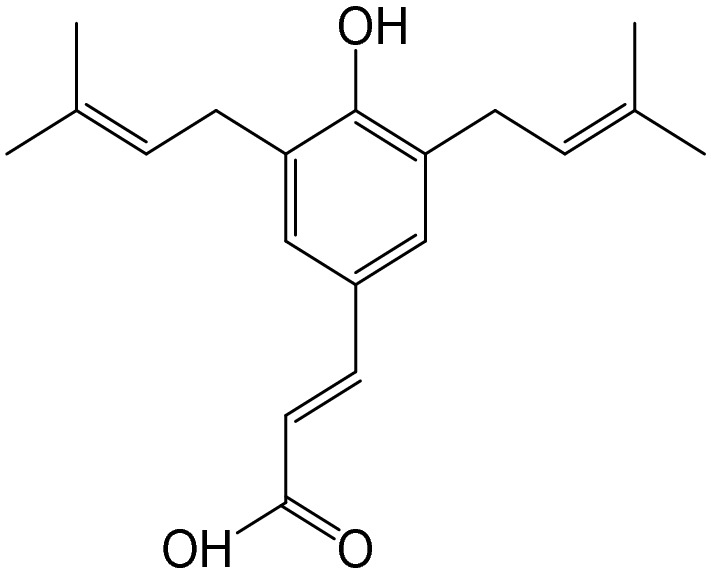
Molecular structure of Artepillin C.

Artepillin C (ArtC; 3, 5-diprenyl-4-hydroxycinnamic acid) (Fig. 1) is the significant biologically active phenolic ingredient and a primary assessment criteria for quality control in Brazilian green propolis^10–12^. ArtC have been also reported to exhibit a wide variety of pharmacological functions, including superior anti-inflammatory, anti-tumor, antimicrobial and antioxidant activities^13–15^ and so on. However, the pharmacological data in vivo showed that ArtC’s absorption efficiency and bioavailability in rat serum were low^16^, which appears to be an inconsistency between the low absorption rate of oral ArtC and its broad biological activity in the body. Thus, it is necessary to further investigate the detailed pharmacokinetic parameters and transport mechanism of ArtC in vitro.

When a bioactive molecule enters the blood circulation system, it could be bound and transported by plasma proteins in the human circulatory system^17^. Human serum albumin (HSA) accounts for 60% of the total plasma protein, and is absolutely abundant in human body^18^. Thus, the exogenous bioactive compounds can be delivered through HSA to the corresponding action target receptors^19–21^. HSA consists of three homologous domains (I, II and III), each of which can be further divided into two subdomains (A and B). There are two major different binding sites, which are located in the hydrophobic cavities of subdomains IIA and IIIA, respectively^22^. The potential binding site of bioactive compound in HSA is necessary to be interpreted by competitive experiments.

There have been lots of studies on the binding interactions between HSA and some bioactive components isolated from Brazilian green propolis, such as ferulic acid^23,24^, coumaric acid^25^, cinnamic acid^25^, and caffeic acid phenethyl ester (CAPE)^26^ in other types of propolis. All the interactions are showed as static binding process, suggesting they form a ground state complex between compounds and HSA. The interaction between ArtC and HSA has been recently reported through spectroscopic techniques^27^, while we almost simultaneously further elucidated the detailed mechanism, and obtained the different results.

In this study, we applied multispectroscopic techniques (including fluorescence, UV–Vis absorption, and circular dichroism (CD) spectra etc.), thermodynamic and molecular docking to figure out the physicochemical basis on how ArtC binding with HSA in vitro. The binding characteristics of reported bioactive components (including ArtC recently) from propolis were also further compared. To sum up, revealing the characteristic interactions between ArtC and HSA in vitro will makes up for and confirm the theoretical fact about ArtC binding and transporting at the physicochemical level in the body. It also provides further insights into the differences in the action of various bioactive compounds from Brazilian green propolis in vivo.

## Materials and methods

### Materials

Human serum albumin (fatty acid free; catalogue no. A-1653; purity > 97%) was purchased from Sigma-Aldrich, Inc. (St. Louis, MO) and used without further purification. All HSA solutions were prepared in pH 7.4 Phosphate-Buffered Saline (PBS) buffer solutions, and kept at − 20 ℃ without light. Artepillin C (ArtC; 3, 5-diprenyl-4-hydroxycinnamic acid) (Fig. 1, purity > 99%) was purchased from Wako Pure Chemical Industries, Ltd (Japan). The concentration of ArtC stock solution was 1.0 × 10^−4^ mol L^−1^ using methanol as the solvent.

### Apparatus and methods

1. Fluorescence spectra were scanned by using a RF-5301PC type fluorospectrophotometer (Shimadzu, Japan) equipped with xenon lamp source and 1.0 cm × 1.0 cm × 4.0 cm quartz cell at four different temperatures (290, 295, 300 and 305 K). The working concentration of HSA was 5.0 × 10^−7^ mol L^−1^, and quantified by the normal BCA method of protein. The widths of excitation and emission slit were set at 5.0 nm. Based on the optimal scanning conditions^28,29^, excitation wavelength was 282 nm, and the emission spectra were recorded between 290 and 500 nm. The maximum emission spectra were observed at 340 nm. The synchronous fluorescence spectra of HSA were measured by increasing concentration of ArtC, by setting Δ = 60 nm and Δ = 15 nm for tryptophan and tyrosine residues, respectively. A thermostat water-bath 9112 was purchased from PolyScience (USA) for controlling the experimental temperature.
2. UV–Vis absorption spectra measurements were recorded on a UV-1800 type spectrophotometer (Shimadzu, Japan) in the wavelength range of 190–400 nm with a 1.0 cm quartz cell at room temperature. The working concentration of both HSA and ArtC was equally 5.0 × 10^−7^ mol L^−1^.
3. The CD spectra were recorded by J-815 CD spectrophotometer (JASCO, Japan) at room temperature, the slit width was set at 5 nm, the speed of scanning was 100 nm/min. Within a 1 cm quartz cell, the ArtC (final concentrations from a to d of 0, 1.5, 2.5, and 5.0 × 10^−6^ mol L^−1^, respectively) was titrated into the HSA solutions with a concentration of 5.0 × 10^−7^ mol L^−1^.
4. Docking study of the binding mode between ArtC and HSA was performed by Molegro Virtual Docker 4.2 software (free trial). The 3D crystal structure of HSA was generated on SWISS-MODEL Workspace^30^. The 3D structure of ArtC was downloaded from the PubChem database of NCBI (CID 5472440, https://pubchem.ncbi.nlm.nih.gov). The best binding pose was obtained according to the searching algorithm of MolDock optimizer and energetic evaluation of the complex with MolDock. The binding pose was then analyzed and displayed by LigPlot + 1.4.5^31^ and displayed by PyMOL software 1.3.x^32^, respectively.
5. Competitive binding assay. To determine the actual binding site of ArtC on HSA, a competitive binding assay was performed referred to the previous studies^26^. The working solutions of warfarin and ibuprofen were separately diluted with methanol to a working solution (1.0 × 10^−4^ mol L^−1^) beforehand. Firstly, when the molar concentration ratio of ArtC to HSA in the experimental system was set to 1:1, the working solutions of warfarin and ibuprofen were then titrated into the complex system, respectively. Secondly, according to the fluorescent decrease of ArtC–HSA complex of each drug, the molar ratio of warfarin to HSA was set to a gradient ratio of 0:1, 1:1, 2:1, 3:1, and 4:1. The ArtC solution was then titrated separately into the corresponding gradient system. All binding constants under the corresponding gradient system were calculated and compared to determine the competitive effects and binding sites.

## Results

### Fluorescence quenching spectra

**Figure 2:**
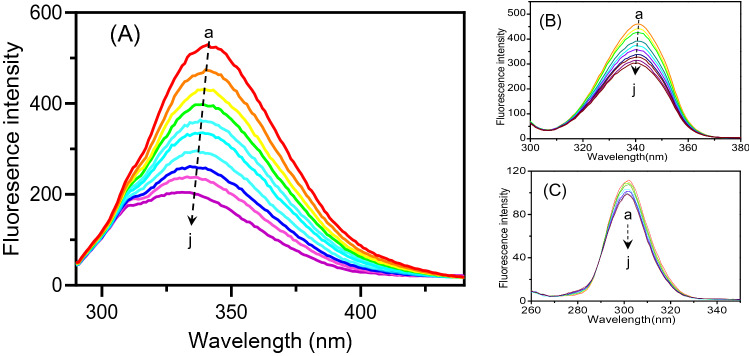
Fluorescence quenching spectra (**A**) and synchronous fluorescence spectra including Δ = 60 nm (Trp) (**B**) and Δ = 15 nm (Tyr) (**C**). The chemical structure of 3, 5-diprenyl-4-hydroxycinnamic acid (Artepillin C, ArtC) (**A**, inset). The fluorescence intensity of HSA decreases as the concentration of ArtC increases. The change of the peak shape is not obvious and the maximum emission wavelength is slightly blue-shifted when ArtC titrated. The concentrations of ArtC from *a* to *j* are 0, 0.05, 0.1, 0.15, 0.2, 0.25, 0.3, 0.35, 0.4, 0.45, and 0.5 × 10^−6^ mol L^−1^, respectively.

Due to the presence of fluorescent amino acid residues, HSA has a fluorescence emission spectrum and exhibits a strong fluorescent peak at 340 nm upon excitation at 282 nm. With increasing ArtC concentration, the fluorescence emission spectra of HSA decreased regularly, and the maximum emission wavelength and the shape peaks seem to shift toward blue of at least 10 nm (Fig. 2A). The results showed that fluorescence quenching of HSA by ArtC was observed during the interaction process.

In this study, synchronous fluorescence spectra were used to observe changes in HSA conformation after ArtC added. When the scanning interval Δλ (Δλ = λ_em_ − λ_ex_) is fixed at 60 and 15 nm, respectively, the experiment provides characteristic information of the tryptophan (Trp) and tyrosine (Tyr) residues, respectively^33^. As shown in Fig. 2, the synchronous fluorescence spectra of the Trp residue in HSA (Fig. 2B) had a great stronger fluorescence emission intensity and larger blue shift of maximum emission wavelength than the Tyr residues in HSA (Fig. 2C). Besides, the shape and blue shift of the synchronous spectra of Trp residue (Fig. 2B) were similar to the spectra of HSA (Fig. 2A), indicating that the interaction site of ArtC should be close to the unique Trp residue in HSA.

### Binding mechanism

The fluorescence quenching process of binding interaction is always divided into static and dynamic (collisional) quenching. Dynamic or static quenching can be distinguished by their differential dependence on temperature and viscosity. Higher temperature results in faster diffusion and hence greater extent of dynamic quenching, whereas the effect is typically reversed for static quenching^34^. The well-known Stern–Volmer equation describes static and dynamic processes^35^ as follows:

1 $$\frac{{F_{0} }}{F} = 1 + K_{q} \tau_{o} [Q] = 1 + K_{sv} [Q]$$

**Figure 3:**
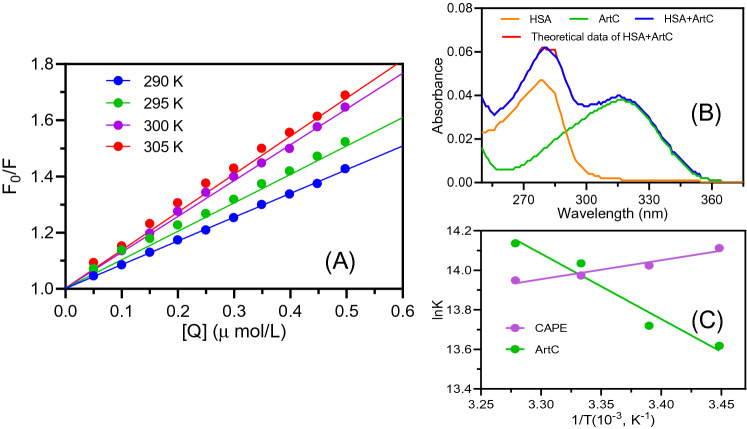
Thermodynamic mechanism analysis. (**A**) The Stern–Volmer plots (at 290, 295, 300, and 305 K). (**B**) Additivity measurement of UV absorption spectra. The theoretical additive (red line) of HSA and ArtC almost coincides with the experimental UV spectrum (blue line) of the mixture (ratio of 1:1) of them. The UV spectra of HSA (orange line) and ArtC (green line) alone with the same concentration of 1.0 × 10^−6^ mol L^−1^ are also shown. (**C**) Comparison of ArtC (this study) and CAPE^31^ of linear regression plot of lnK versus 1/T based on the values of Δ*H* and Δ*S* (at 290, 295, 300, and 305 K).

In this equation, *F*_0_ and *F* are the fluorescence intensities in the absence and presence of quencher, respectively; [*Q*] is the quencher concentration, *K*_*q*_ is the bimolecular quenching rate constant, and *K*_*sv*_ is the Stern–Volmer quenching constant. In the measurements of different temperatures, all *K*_*sv*_ values (listed in Table 1) increased (Fig. 3A) with the rising of the experimental temperatures. As the rising temperature can accelerate the collision speed of compounds, then speed up the dynamic binding process^35^, the results indicated that the fluorescence quenching mechanism of HSA to ArtC is dynamic process. However, Table 1 showed that the values of *K*_q_ are much higher than the maximum diffusion collision quenching rate constant (2.0 × 10^10^ L mol^−1^ s^−1^), suggesting that static quenching occurred in HSA-ArtC system.

**Table 1:** Fluorescence quenching constants (in equation of Stern–Volmer) for the interaction between Artepillin C and HSA.

*T*(*K*)	*K*_*sv*_*/*(L mol^−1^)	*K*_*q*_/(L mol^−1^ s^−1^)	*R*^2^
290	0.842 × 10^6^	0.842 × 10^14^	0.9993^a^
295	0.985 × 10^6^	0.985 × 10^14^	0.9988^a^
300	1.261 × 10^6^	1.261 × 10^14^	0.9974^a^
305	1.321 × 10^6^	1.321 × 10^14^	0.9982^a^

^a^Correlation coefficient.

In addition, as well known, whether new complex can be used to distinguish between static and dynamic quenching mechanisms, and UV absorption spectra of complicated biological system exhibit the additivity if all components do not react and produce new complex^25^. Therefore, to further confirm the binding mechanism, we measured the additivity of UV absorption spectra of HSA and ArtC. As seen in Fig. 3B, the theoretical additive UV spectrum of HSA and ArtC almost coincided with the experimental UV spectrum of HSA-ArtC mixture (ratio of 1:1), indicating that no new complex was produced when ArtC added into HSA solutions. This result was evidently different from the static quenching process that the production of new complexes^35^, thus confirming that the quenching process of HSA and ArtC should be collisional dynamic quenching rather than static quenching.

### Thermodynamic analysis and binding forces

In this study, the binding constant (*K*_A_) and the number of binding sites (*n*) can be calculated by the double logarithm regression curve as follow^36^:

2 $$\lg \frac{{F_{0} - F}}{F} = \lg K_{A} + n\lg [Q]$$

where *F*_0_ is the fluorescence intensity in the absence of a quencher and *F* is the fluorescence intensity in the presence of a quencher at concentration [*Q*]. *K*_*A*_ is the apparent association constant, and n is the number of binding sites per protein. As show in the Table 1, the *K*_*A*_ values increase with the temperature rising. According to Eq. (2), the binding sites *n* was close to 1 (0.8430–0.9667) (Table 2), which indicate that the quencher ratio is about 1:1.

**Table 2:** Thermodynamic analysis and binding forces of the interaction between Artepillin C and HSA.

*T* (*K*)	*K*_*A*_ (L mol^−1^)	*n*	*R*^2^	Δ*H* (kJ mol^−1^)	Δ*S* (J mol^−1^ K^−1^)	Δ*G* (kJ mol^−1^)	Binding force
290	0.821 × 10^6^	0.9667	0.9993^a^	− 27.507	207.877	− 32.836	Hydrophobic forces and electrostatic force
295	0.908 × 10^6^	0.8430	0.9973^a^			− 33.650	
300	1.246 × 10^6^	0.9541	0.9988^a^			− 35.008	
305	1.378 × 10^6^	0.9493	0.9914^a^			− 35.849	

^a^Correlation coefficient.

In general, the binding interaction between organic compound and macromolecular proteins mainly includes electrostatic forces, hydrogen bonds, hydrophobic interaction, Van der Waals forces and so on^37^. These thermodynamic interaction modes could be estimated by the various thermodynamic conditions as follows: (1) Δ*H* > 0 and Δ*S* > 0, hydrophobic forces; (2) Δ*H* < 0 and Δ*S* < 0, van der Waals interactions and hydrogen bonds; (3) Δ*H* < 0 or Δ*H* ≈ 0 and Δ*S* > 0, hydrophobic forces and electrostatic force^36,38^. These thermodynamic parameters can be obtained from the thermodynamic equations below:

3 $$\Delta G = - RT\ln K = \Delta H - T\Delta S$$

4 $$\ln K = - \frac{\Delta H}{{RT}} + \frac{\Delta S}{R}$$

where Δ*G*, Δ*H*, and Δ*S* are the free energy change, enthalpy change, and entropy change, respectively; *R* is the gas constant; *T* is the experimental temperature and the *K* is the binding constant at the respective temperatures. Based on the formulas (3) and (4), the parameters were calculated as Δ*G* < 0, Δ*H* =  − 27.507 kJ mol^−1^, and Δ*S* = 207.877 J mol^−1^ K^−1^ (Table 2). Here, Δ*G* < 0 indicates that all interactions are spontaneous in all four temperatures. As Δ*S* is much larger than zero and Δ*H* is slightly lower than zero, it suggests that hydrophobic interaction is more significant force than electrostatic force in the process of ArtC binding with HSA. Moreover, their linearized thermodynamic curves are also clearly different shown in Fig. 3C. This implies that the complex Brazilian green propolis components have diverse binding forces that interact with HSA.

### Fluorescence resonance energy transfer (FRET) analysis

Since ArtC quenches the intrinsic fluorescence of HSA with a dynamic quenching process, the interaction distance between them can be determined by fluorescence resonance energy transfer (FRET) theory^39^. FRET theory mainly requires three conditions: (1) the necessary donor and acceptor dipoles, (2) the fluorescence emission spectrum of the donor should partly overlap with the absorption spectrum of the acceptor, and (3) the distance between the donor and acceptor should be shorter than 10 nm. The theory was used to elucidate the binding interaction between HSA (donor) and ArtC (acceptor) in this study based on the following equations^40^:

5 $$E = \frac{{R_{0}^{6} }}{{R_{0}^{6} + r_{0}^{6} }} = 1 - \frac{F}{{F_{0} }},$$

6 $$R_{0}^{6} = 8.8 \times 10^{ - 25} K^{2} N^{ - 4} \varphi J,$$

7 $$J = \frac{{\sum {F(\lambda )\varepsilon (\lambda )\lambda^{ - 4} \Delta \lambda } }}{{\sum {F(\lambda )\Delta \lambda } }}.$$

**Figure 4:**
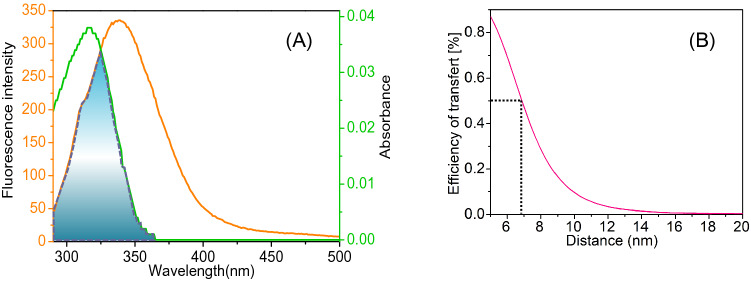
Fluorescence resonance energy transfer (FRET) analysis. Overlapping of fluorescence spectra of HSA and UV absorption spectra of ArtC (**A**), and their evolution of ET (efficiency of transfer) (**B**). The binding distance *r*_*0*_ is shown as about 7.05 nm when the value of ET (%) reaches 50%. *c* (HSA) = *c* (ArtC) = 1 μmol L^−1^; pH 7.4; λ_ex_ = 282 nm.

Here, the efficiency of energy transfer (*E*) is related to the distance *R*_0_ between the donor and the acceptor. *R*_0_ is the critical distance at which the efficiency of transfer reaches 50%^39^. *r*_0_ is the binding distance between the donor and the receptor, *K*^2^ is the spatial orientation factor of the dipole, *N* is the refractive index of the medium, and *Φ* is the fluorescence quantum yield of HSA, and *J* is the overlap integral of the fluorescence emission spectrum of the donor with the absorption spectrum of the acceptor. In this study, *K*^2^ = 2/3; N = 1.336; *Φ* = 0.118^41^. By Eqs. (5)–(7), according to the overlapping of fluorescence spectra of HSA and UV absorption spectra of ArtC (Fig. 4A), as well as their evolution of ET (efficiency of transfer). As shown in Fig. 4B, the binding distance *R*_0_ is calculated and shown as 6.88 nm when ET reaches 50%, and *r*_0_ is then calculated as 7.05 nm. Since *r*_0_ is less than 10 nm^40^, it indicates that the fluorescence quenching from HSA to ArtC is actually caused from the fluorescence energy transfer from HSA to ArtC, which should be in accord with the FRET theory.

### Molecular docking analysis

**Figure 5:**
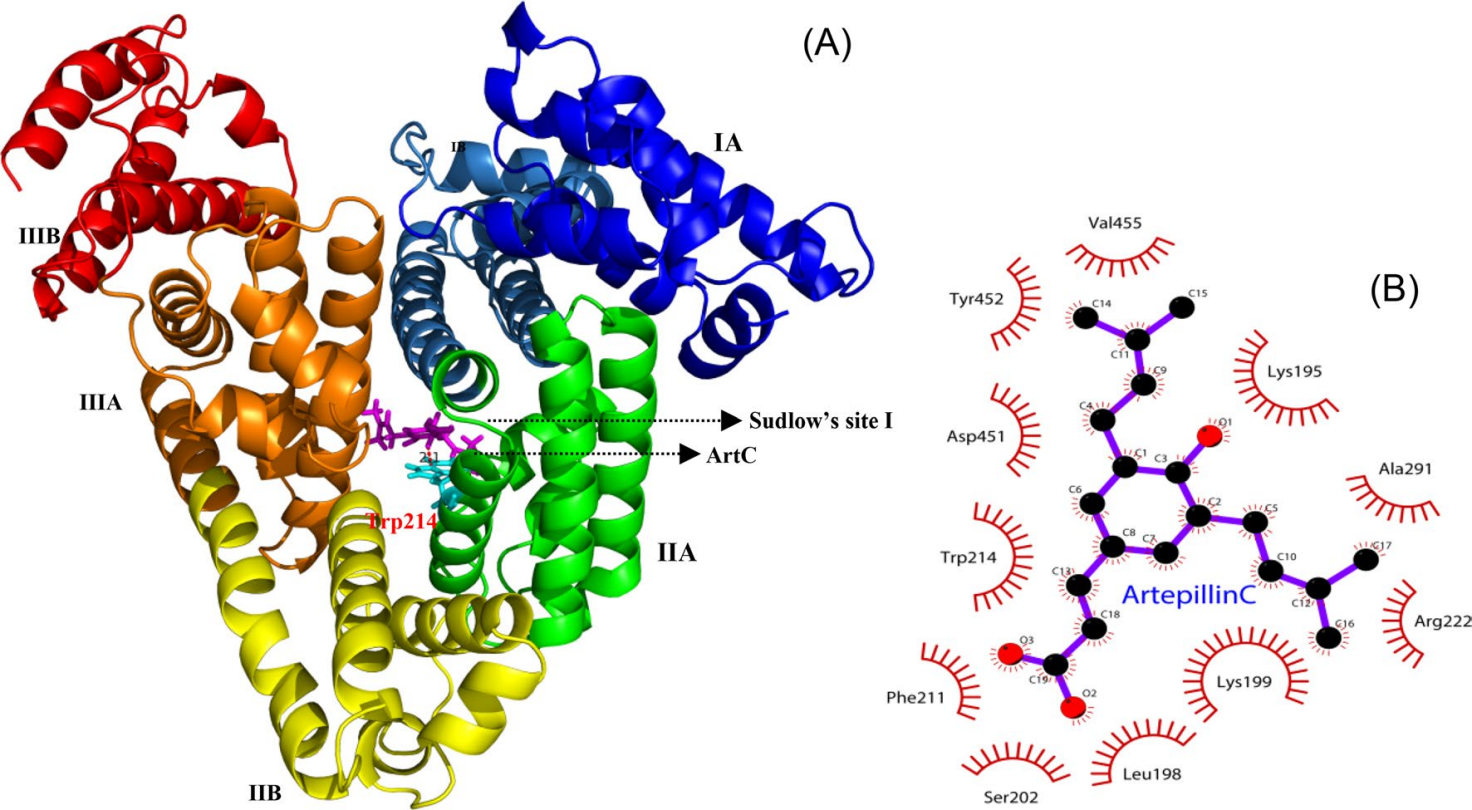
Molecular docking analysis. (**A**) ArtC (plum) interacts with residues of HSA located on α-helices in subdomain IIA of HSA. The cyan residue represents Trp214 that having a distance of 2.1 nm from ArtC. (**B**) shows the detailed contributions of 11 significant residues of HSA that interact with ArtC.

In order to elucidate and describe the exquisite process of HSA binding with ArtC in detail, the molecular docking analysis was performed by using MVD software. According to the running results of Rerank Score, the potential binding cavity and the optimized pose were predicted and obtained. The docking results were finally exhibited by Ligplot + and Pymol software^30,31^. As seen in Fig. 5A, ArtC seems to be located in the Sudlow’s site I (FA7), which is in the IIA site of HSA. Interestingly, the only tryptophan Trp^214^, expected to be 2.1 nm from ArtC. This is almost in line with the calculation of FRET in “Fluorescence resonance energy transfer (FRET) analysis” section of this study above. In order to elucidate the detailed amino acids of HSA interacting with ArtC, a two-dimensional interaction diagram was drawn. As seen in Fig. 5B, it was found that ArtC was involved in a binding cavity which was comprised of 11 amino acid residues of HSA. Based on the biochemical characteristics of these residues, it is predicted that four hydrophobic residues (Leu^198^, Phe^211^, Ala^291^, and Val^455^) mainly contribute the hydrophobic interactions between HSA and ArtC. However, the other seven hydrophilic residues may be involved in the electrostatic force interactions. The two fluorescence residues Trp^214^ and Tyr^452^ are also close to ArtC, indicating that the two residues are likely to participate in the binding interaction and the energetic transformation. This is in accordant with the synchronic fluorescence spectra in “Fluorescence quenching spectra” section of this study.

### Circular dichroism (CD) spectra

**Figure 6:**
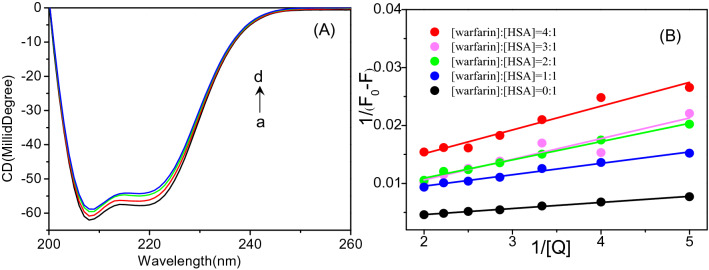
Conformational change and binding site analysis. (**A**) Circular dichroism (CD) spectra of HSA and ArtC. With the addition of ArtC (final concentrations from a to d of 0, 1.5, 2.5, and 5.0 × 10^−6^ mol L^−1^, respectively), the typical shoulder peaks of α-helix increase at 209 and 222 nm. (**B**) Determination of the ArtC binding site in HSA by a warfarin-based competitive assay. It is evident that the larger of the molar ratio of warfarin–HSA (a serial of gradient ratio as 0:1, 1:1, 2:1, 3:1, 4:1 and 5:1), the higher of the corresponding slopes. It suggests that ArtC effectively competes with warfarin on the binding site, Sudlow’s site I of subdomain IIA of HSA.

CD spectroscopic analysis is always effective for monitoring secondary structure changes in proteins when interacting with ligands^42^. As seen in Fig. 6A, when ArtC was titrated into the HSA solution, two negative shoulder peaks of the HSA proteins were observed at wavelengths of 208 and 222 nm, and the *θ* values of the two peaks were significantly increased. Based on the original *θ* values of the two peaks, the percentage of α-helices in the two-dimensional structure of HSA is calculated^37^. Compared with the wide molecular ratio range of HSA to ArtC (from 1:0, 1:3, 1:5 to 1:10), the change of percentage of HSA α-helix was not very large (from 72.2%, 70.3, 67.5 to 66.8%, respectively). It indicates that ArtC could not significantly affect the structure of HSA.

### Determination of binding site of ArtC in HSA

It is believed that warfarin and ibuprofen are located at specific binding sites of HSA, namely I and II of Sudlow’ site^22^. When warfarin and ibuprofen were titrated into equimolar ArtC–HSA systems, warfarin had a more pronounced fluorescence quenching effect on ArtC–HSA complex than ibuprofen (Fig. S1). Therefore, in order to study the competitive conditions between warfarin and ArtC towards HSA, a series of molar ratios of warfarin and HSA were set to 1:1, 2:1, 3:1 and 4:1. According to the following Lineweaver–Burk equation (Eq. (8)), the binding constants of the corresponding ArtC and warfarin-HSA complexes were measured and calculated.

8 $$\frac{1}{{F_{0} - F}} = \frac{1}{{F_{0} }} + \frac{1}{{K_{A} F_{0} \left[ {\text{Q}} \right]}}$$

where all the parameters are the same to Eq. (2). As seen in Fig. 6B and Table 3, as the warfarin-HSA molar ratio increases, the *K*_A_ value decreases, indicating that ArtC and warfarin compete significantly at the HSA binding site. Therefore, it is speculated that Sudlow’s site I in subdomain IIA is the major binding site for ArtC in HSA, which is consistent with the hypothesis of docking analysis in “Molecular docking analysis” section above.

**Table 3:** Competitive binding assay of ArtC binding to different proportions of HSA-warfarin complex.

[HSA]:[warfarin]	Equations	*R*^2^	*K*_*A*_ (L mol^−1^)
1:0	y = 0.0011x + 0.0025	0.9968^a^	1.74 × 10^6^
1:1	y = 0.002x + 0.0056	0.9878^a^	9.96 × 10^5^
1:2	y = 0.0032x + 0.0046	0.9932^a^	6.63 × 10^5^
1:3	y = 0.0036x + 0.0035	0.9036^a^	6.15 × 10^5^
1:4	y = 0.0041x + 0.0069	0.9628^a^	5.69 × 10^5^

^a^Correlation coefficient.

## Discussion

In this study, we used multispectroscopic techniques to discover a unique dynamic mode interaction between HSA and ArtC, which is a representative bioactive component from Brazilian green propolis. In order to compare and describe the similarities and differences in the characteristic of ArtC and other reported propolis components, we listed all of their relevant interaction parameters. As seen in Table 4, in all reported propolis components, ArtC exhibits the second highest binding constant (9.08 × 10^5^ L mol^−1^), only lower than that of CAPE (2.15 × 10^6^ L mol^−1^). Most interestingly, as one of the most significant components in Brazilian green propolis^10^, the binding interaction between ArtC and HSA is unique dynamic mode, which is evidently different with other propolis components.

**Table 4:** Comparison of the characteristics of ArtC and other propolis components based on the fluorescence quenching spectra.

Propolis components	*K*_*A*_/(L mol^−1^)	Binding mechanism	Binding distance (nm)	Cavity of HSA
ArtC	9.08 × 10^5^ (in this study)	Dynamic	3.75	IIA
	2.99 × 10^4^^27^	Static	–	IIA
CAPE	2.15 × 10^6^^26^	Static	5.7	IIA
Caffeic acid	4.31 × 10^5^^24^	Static	–	IIA^a^
	1.60 × 10^5^^24^	Static	2.79	–
Ferulic acid	4.72 × 10^5^^23^	Static	–	IIA^a^
	2.23 × 10^4^^23^	Static	3.57	IIIA^b^
*p*-coumaric acid	1.10 × 10^5^^25^	Static	2.59	–
Chlorogenic acid	4.37 × 10^4^^23^	Static	2.45	IIA^b^
Cinnamic acid	4.00 × 10^3^^25^	Static	1.87	–

^a^The binding site was predicted based on the fluorescence quenching both the emission and synchronous spectra of HSA-ligand.

^b^The binding site was predicted based on the fluorescence quenching fraction (%) of HSA-ligand complex.

Stern–Volmer plot indicated that HSA-ArtC seemed to be the dynamic quenching mechanism, due to the increasing of *K*_*sv*_ values with the rising of temperature. However, Table 1 showed that the values of *K*_*q*_ are much higher than the maximum diffusion collision quenching rate constant (2.0 × 10^10^ L mol^−1^ s^−1^), suggesting that static quenching occurred in HSA-ArtC system. In order to confirm the quenching mechanism, UV spectra were performed. As seen in Fig. 3B, since the theoretical additive UV spectrum of HSA and ArtC almost coincided with the experimental UV spectrum of HSA-ArtC mixture. It suggested that there were not new complex produced when ArtC added into HSA solutions, which is in accordance with the colliding characteristics involved in the dynamic quenching^35^. Therefore, we synthesize the consideration that the binding interaction of HSA-ArtC should be the dynamic quenching mechanism. However, our results were opposite to the simultaneously similar study of ArtC binding with HSA^27^ (Table 4). The difference between each other may be related to the material source of ArtC, which was isolated from n-hexane extract of the Brazilian green propolis^27^, and directly bought from commercial company in this study, respectively. This controversy needs to be further investigated in the future.

The pharmacological data in vivo showed that ArtC exhibited the low concentration in rat serum. For example, compared with another propolis component such as *p*-coumaric acid, the maximum concentration of *p*-coumaric acid in the portal vein was 29.36 ± 2.83 μmol L^−1^, while ArtC was only 3.64 ± 0.64 μmol L^−1^. In the abdominal artery, *p*-coumaric acid was 30.43 ± 1.94 μmol L^−1^, while ArtC was only 0.31 ± 0.09 μmol L^−1^^16^. This low absorption efficiency and low bioavailability of ArtC in serum may be due to the unique dynamic binding interaction with HSA reported in this study. In contrast, most propolis ingredients such as *p*-coumaric acid exhibit the static binding interaction as shown above (Table 4). The static interaction might protect these organic compounds from elimination in the body due to the conjugation with HSA.

Although the dynamic mode is quite different from the static mode of all other reported propolis components, the blue shift of fluorescence quenching spectra of HSA by ArtC indicates that the polarity of the microenvironment decreased with the addition of ArtC close the Trp residues (Fig. 2B), which is similar to the other interactions between plasma proteins and chemical drugs as well as organic compounds^37,43,44^. Moreover, the quencher ratio is about 1:1, which is in accordance with the previous reports about drugs^42,45^. It suggests that despite the different binding mechanism, the quenching microenvironment of the interaction between fluorescence amino acid and ArtC remains similar.

The thermodynamic analysis showed that the hydrophobic interaction is more significant force than electrostatic force in the process of ArtC binding with HSA (Table 2). It is in accordance with the binding interaction between some flavonoids and BSA^46^. While it is different from another propolis component, CAPE, which is mainly driven by hydrogen bonds and van der Waals forces^26^.

Considering the binding distance and binding site, the competitive experiments showed that ArtC existed in subdomain IIA, which represents Sudlow’s site I and is a preferential binding site for bulky heterocyclic anions^29^ (e.g. warfarin), while subdomain IIIA represents Sudlow’s site II, which is preferred by aromatic carboxylates with an extended conformation (e.g. ibuprofen)^47^. In particular, ArtC also shows a quite similar to other compounds, the IIA site is indeed a common binding site for most propolis components including CAPE^26^, caffeic acid^25^, chlorogenic acid^23^ and ferulic acid^24^ (Table 4).

In summary, this unique physicochemical binding mechanism between ArtC and HSA determines its mode of transport in the body, which in turn may promote its unique biological and physiological functions. This also implies the need to consider the transport of the body in the study of the biological activity of other representative functional components of Brazilian green propolis.

## Supplementary information

Supplementary Information.
