# Supplementary material for: Unique dynamic mode between Artepillin C and human serum albumin implies the characteristics of Brazilian green propolis representative bioactive component
Source: Sci Rep. 2020 Oct 14;10:17277. doi: 10.1038/s41598-020-74197-4 (PMC7560867; doi:10.1038/s41598-020-74197-4)
Supplement: Supplementary file 1 — Supplementary Information. [file 41598_2020_74197_MOESM1_ESM.docx]

Unique dynamic mode between Artepillin C and human serum albumin implies the characteristics of Brazilian green propolis representative bioactive component

Fan Wu ^a^, Xin-Mi Song ^a^, Yi-Lei Qiu ^a^, Huo-Qing Zheng ^b^, Fu-Liang Hu ^b^, and

Hong-Liang Li ^a, *^

*a Zhejiang Provincial Key Laboratory of Biometrology and Inspection & Quarantine,* *College of Life Sciences,* *China Jiliang University,* *Hangzhou 310018,* *China*

*b College of Animal Sciences,* *Zhejiang University, Hangzhou 310058,China*

* Corresponding authors at: College of Life Sciences, China Jiliang University, Hangzhou 310018, China, Tel./Fax: +86 571 86835774

E-mail address: [hlli@cjlu.edu.cn](mailto:hlli@cjlu.edu.cn) (H.-L. Li)

Figure S1. Competitive binding assay of warfarin and ibuprofen separately binding with HSA-ArtC complex. Warfarin significantly declined the fluorescence intensity of HSA-ArtC complex, compared with ibuprofen.

[HSA+ArtC] + ibuprofen

[HSA+ArtC] + warfarin
